# Kidney tubular injury induced by valproic acid: systematic literature review

**DOI:** 10.1007/s00467-022-05869-8

**Published:** 2023-01-16

**Authors:** Giulia Anguissola, Dennis Leu, Giacomo D. Simonetti, Barbara Goeggel Simonetti, Sebastiano A. G. Lava, Gregorio P. Milani, Mario G. Bianchetti, Martin Scoglio

**Affiliations:** 1 Pediatric Institute of Southern Switzerland, Ente Ospedaliero Cantonale, Bellinzona, Switzerland; 2 Faculty of Biomedical Sciences, Università Della Svizzera Italiana, Lugano, Switzerland; 3 Department of Neurology, Inselspital, University Hospital and University of Bern, Bern, Switzerland; 4 Pediatric Cardiology Unit, Department of Pediatrics, Centre Hospitalier Universitaire Vaudois and University of Lausanne, Lausanne, Switzerland; 5 Clinical Pharmacology and Therapeutics Group, University College London, London, UK; 6 Pediatric Unit, Fondazione IRCCS Ca’ Granda Ospedale Maggiore Policlinico, Milan, Italy; 7 Department of Clinical Sciences and Community Health, Università Degli Studi Di Milano, Milan, Italy

**Keywords:** Fanconi syndrome, Kidney tubular damage, Mitochondrial toxicity, Valproic acid

## Abstract

**Background:**

Valproic acid is prescribed for epilepsy and as prophylaxis for bipolar disorder and migraine headaches. It has also been implicated as a cause of a kidney tubular injury.

**Methods:**

We undertook a review of the literature to characterize the biochemical and histopathological features of the overt kidney tubular injury and to evaluate the possible existence of a pauci-symptomatic injury. The pre-registered review (CRD42022360357) was performed following the Preferred Reporting Items for Systematic Reviews and Meta-Analyses (PRISMA) methodology. Searches were conducted in Excerpta Medica, the National Library of Medicine, and Web of Science. The gray literature was also considered.

**Results:**

For the final analysis, we retained 36 articles: 28 case reports documented 48 individuals with epilepsy on valproic acid for 7 months or more and presenting with features consistent with an overt kidney tubular injury. The following disturbances were noted: hypophosphatemia (*N* = 46), normoglycemic glycosuria (*N* = 46), total proteinuria (*N* = 45), metabolic acidosis (*N* = 36), hypouricemia (*N* = 27), tubular proteinuria (*N* = 27), hypokalemia (*N* = 23), and hypocalcemia (*N* = 8). A biopsy, obtained in six cases, disclosed altered proximal tubular cells with giant and dysmorphic mitochondria. Eight case series addressed the existence of a pauci- or even asymptomatic kidney injury. In the reported 285 subjects on valproic acid for 7 months or more, an isolated tubular proteinuria, mostly N-acetyl-β-glucosaminidase, was often noted.

**Conclusions:**

Valproic acid may induce an overt kidney tubular injury, which is associated with a proximal tubular mitochondrial toxicity. Treatment for 7 months or more is often associated with a pauci- or oligosymptomatic kidney tubular injury.

**Graphical abstract:**

**Figure:**
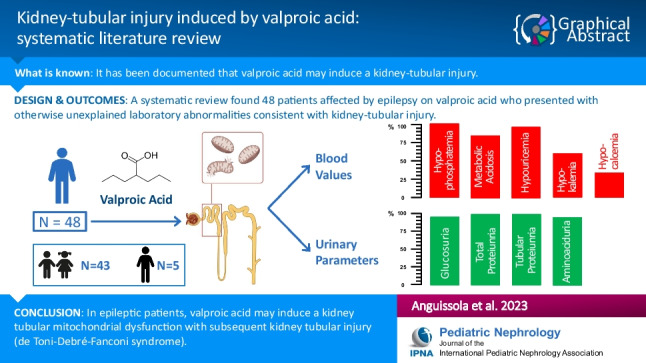
A higher resolution version of the Graphical abstract is available as Supplementary information

**Supplementary information:**

The online version contains supplementary material available at 10.1007/s00467-022-05869-8.

## Introduction

Valproic acid, a branched-chain carboxylic acid, was introduced as an anti-epileptic agent 50 years ago [1]. It is currently prescribed also as prophylaxis for bipolar disorder and migraine headaches [2]. While generally considered safe, valproic acid is associated with some adverse effects. These include skin reactions, bone marrow suppression, altered liver or pancreatic enzymes, hyperammonemia, carnitine deficiency, and a tendency to malformation development after in utero exposure [3].

Valproic acid has also been implicated as a cause of an overt kidney tubular injury since 1981 [3, 4]. However, the characteristics of the kidney tubular injury associated with valproic acid have never been comprehensively evaluated. The purposes of this systematic review of the literature were to characterize the biochemical and histopathological features of the overt kidney tubular injury (and its predisposing factors), to evaluate the possible existence of a pauci-symptomatic kidney tubular injury, and to speculate on the mechanisms that underlie the injury.

## Methods

### Data sources–searches

This work was registered at the International Prospective Register of Systematic Reviews (CRD42022360357) and undertaken in agreement with the 2020 edition of the Preferred Reporting Items for Systematic Reviews and Meta-Analyses (PRISMA) methodology [5]. Excerpta Medica, the National Library of Medicine, and Web of Sciences were used for a systematic literature search with no date or language limits for the terms (“valproate” OR “valproic acid”) AND (“acidosis” OR “dyselectrolytemia” OR “Fanconi syndrome” OR “tubular disorder” OR “tubular proteinuria” OR “tubulopathy”). The gray literature, articles already known to the authors, and references listed within bibliographies were also considered for inclusion. The searches were performed in April 2022 and repeated before submission.

### Article selection–data extraction–classification

Of interest were original articles and letters reporting individually documented humans given valproic acid presenting with otherwise unexplained laboratory findings consistent with an overt kidney tubular injury. Also eligible were case series addressing the possible existence of a pauci-symptomatic kidney tubular injury associated with the use of valproic acid [6–8]. In a first round, the results of the initial literature were screened based on titles and abstracts. In a second round, the full text of the remaining articles was assessed.

Eligible for the overt kidney tubular injury study were individuals on long-term therapy with valproic acid presenting with at least three otherwise unexplained laboratory abnormalities: hypocalcemia or hypercalcemia; hypokalemia or hyperkalemia; hyponatremia or hypernatremia; hypophosphatemia or hyperphosphatemia; hypouricemia or hyperuricemia; metabolic acid–base disturbances (acidosis or alkalosis); generalized aminoaciduria; normoglycemic glucosuria; pathologically increased total protein excretion; increased urinary excretion of a tubular protein (such as N-acetyl-β-glucosaminidase, retinol-binding protein, or β_2_-microglobulin). The concomitant existence of liver or pancreatic damage was also addressed.

Circulating creatinine and aminotransferase levels; the urinary excretion of potassium, phosphate, and uric acid; and the results of kidney biopsy studies were also recorded. The normal range for many laboratory values vary with age, sex, laboratory technique (or equipment), and collection protocol. Hence, the reference values employed for each individual case were used for this analysis. The diagnosis of liver damage was made in cases with a more than two-fold elevation in aminotransferase ratio compared to the laboratory’s own values [3], that of pancreatitis in cases with an increase in amylase or lipase values to three times or greater than the upper normal limit [9]. Of interest were also the underlying condition (with emphasis on congenital metabolic disorders), the dosage, and the duration of the therapy with valproic acid and any co-medication (including carnitine supplementation).

To acquire missing data, attempts were made to contact the original authors. Two authors performed the literature search, the selection of reports retained for analysis, and the data extraction. One author entered the data into a pilot-tested database, and the second author verified the correctness of the data entry. Discrepancies were solved by consensus and, where necessary, a third author was involved.

### Analysis

The accuracy in documenting the clinical and laboratory data and time to recovery of electrolyte and acid–base imbalance was used to grade completeness of reporting as excellent, good, or satisfactory [7]. Pairwise deletion was used to deal with missing data [10]. Quantitative variables are presented as median and interquartile range (or mean and standard deviation) and were analyzed using the Mann–Whitney–Wilcoxon test [11], the Wilcoxon signed rank test [11], the unpaired *t*-test [12] or the paired *t*-test [12], as appropriate. Two-sided *P* values of < 0.05 were considered to indicate significance.

## Results

### Search results

**Fig. 1:**
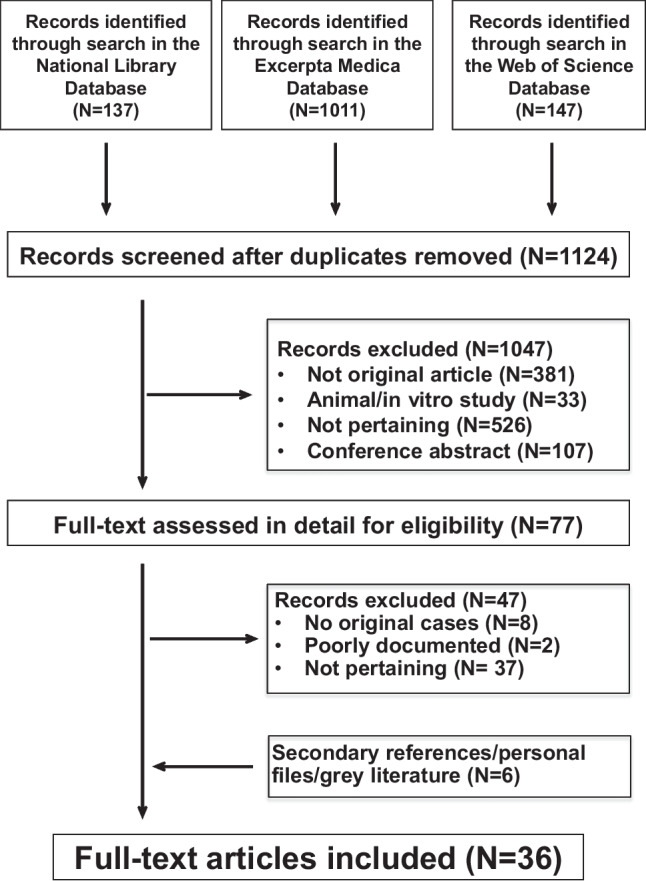
Kidney tubular injury induced by valproic acid. Flowchart of the literature search

The study flowchart is presented in Fig. 1. For the overt kidney tubular injury study, we retained 28 articles [4, 13–39] documenting individuals presenting with three or more laboratory features consistent with a kidney tubular injury. We also found 8 reports [40–47] addressing the possible existence of a pauci-symptomatic kidney tubular damage. The mentioned 36 reports [4, 13–47] were published between 1981 and 2022 in English from the following countries: Japan (*N* = 11), USA (*N* = 8), Türkiye (*N* = 4), UK (*N* = 3), Canada (*N* = 2), Germany (*N* = 2), Italy (*N* = 2), France (*N* = 1), Iran (*N* = 1), Spain (*N* = 1), and Sri Lanka (*N* = 1).

### Findings

#### Overt kidney tubular injury

Three or more otherwise unexplained laboratory abnormalities consistent with an overt kidney tubular injury were found in 48 epileptic subjects on valproic acid for 7 months or more [4, 13–39]. No patient on treatment with valproic acid because of a bipolar disorder or a migraine headache was reported to have otherwise unexplained laboratory abnormalities. Reporting completeness was excellent in 9, good in 28, and acceptable in the remaining 11 cases.

The characteristics of the 48 patients (90% ≤ 16 years of age) with epilepsy are presented in Table 1: most of them very often also suffered from a developmental disability and were on medication with two or more antiepileptic agents other than valproic acid. Pathologically altered liver enzymes were observed in approximately every fifth case.

**Table 1:** Baseline characteristics of 48 epileptic patients 2.0 to 35 years of age with an overt tubular injury on treatment with valproic acid. Results are given as frequency and percentage or as median and interquartile range

Demographics (*N* = 48)	
Female:male ratio	0.92
Age	
Years	7.5 [4.8–12]
≤ 16 years, *N* (%)	43 (90)
Neurologic feature other than epilepsy (*N* = 48)	
Intellectual disability, *N* (%)	6 (13)
Cerebral palsy, *N* (%)	1 (2.1)
Disability and cerebral palsy, *N* (%)	36 (75)
None, *N* (%)	5 (10)
Tube feeding, *N* (%)	31 (65)
Antiepileptic medication (*N* = 48)	
Valproic acid alone, *N* (%)	8 (17)
One more antiepileptic drug, N (%)	15 (31)
Two or more further antiepileptic drugs, *N* (%)	25 (52)
Duration of treatment with valproic acid (*N* = 40)	
≤ 6 months, *N* (%)	0 (0)
7–24 months, *N* (%)	10 (25)
≥ 25 months, *N* (%)	30 (75)
Valproic acid dosage (*N* = 22)	
mg/kg daily	31 [24–38]
≥ 50 mg/kg daily, *N* (%)	3 (14)
Altered liver enzymes, *N* (%)	9 (19)
Altered pancreatic enzymes, *N* (%)	0 (0)

**Fig. 2:**
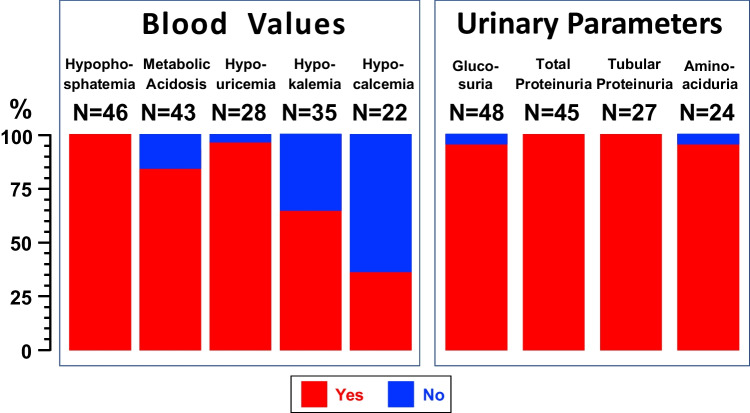
Electrolyte disturbances in blood (left panel) and urinary abnormalities (right panel) observed in 48 epileptic patients 2.0 to 35 years of age with an overt tubular disorder on treatment with valproic acid

Hypercalcemia, hyperkalemia, hyperphosphatemia, and hyperuricemia were never detected. The following electrolyte disturbances were noted in more than five cases (Fig. 2, left panel): hypophosphatemia (*N* = 46), metabolic acidosis (*N* = 36), hypouricemia (*N* = 27), hypokalemia (*N* = 23), and hypocalcemia (*N* = 8). Hypernatremia (*N* = 3), hyponatremia (*N* = 2) and metabolic alkalosis (*N* = 1) were observed only in a few cases. The urinary excretion of phosphate, uric acid, and potassium was inappropriately elevated in 36, 22, and 5 cases, respectively.

The following further urinary abnormalities were observed (Fig. 2, right panel): normoglycemic glycosuria in 46, total proteinuria in 45, and tubular proteinuria in 27 (β_2_-microglobulin, *N* = 25; β_2_-microglobulin and N-acetyl-β-glucosaminidase, *N* = 1; retinol-binding protein, *N* = 1), and generalized aminoaciduria in 23 cases. Finally, the circulating creatinine level was pathologically increased in three cases [16, 19, 37].

A kidney biopsy was obtained in the three cases with an increased circulating creatinine [16, 19, 37], as well as in three out of the remaining 45 cases [4, 14, 38]. It was evaluated by light microscopy alone in one case [16] and also by electron microscopy in the remaining five cases [4, 14, 19, 37, 38]. By light microscopy, the proximal tubular cells demonstrated eosinophilic inclusions consistent with giant mitochondria in three cases. Altered proximal tubular epithelial cells with focal giant mitochondria and numerous dysmorphic mitochondria exhibiting abnormal shapes, sizes, and orientation of cristae were disclosed by electron microscopy in 4 out of 5 cases.

The treatment with valproic acid was stopped in all 48 cases. Sixteen cases were also prescribed a carnitine supplementation. The time to resolution of the laboratory abnormalities was documented in 35 cases: it was < 4 months in 15, ranged between 4 and 6 months in 11, and between 7 and 12 months in the remaining nine cases. A similar (*P* = 0.78) time to resolution was noted in patients with (*N* = 8; 5 (3–10) months) and without (*N* = 27; 4 (3–7) months) carnitine supplementation.

#### Pauci-symptomatic kidney tubular injury

Eight reports [40–47] investigated the possible existence of a pauci-symptomatic kidney tubular injury in 285 epileptic children on valproic acid. The mentioned reports provided information on 5 cross-sectional [43–47] and 4 longitudinal [40–42, 46] studies.

Altunbasak et al. [43] investigated 15 children on valproic acid for at least 6 months. Circulating creatinine, uric acid, and inorganic phosphate, and the urinary excretion of phosphate and β_2_-microglobulin were similar in patients and controls. On the contrary, the urinary N-acetyl-β-glucosaminidase was higher (*P* = 0.001) in patients.

Havali et al. [46] performed two studies. In a cross-sectional study, the authors investigated 30 children on valproic acid for 3 to 24 months. Creatinine and cystatin were not increased in children on valproic acid as compared with 26 healthy children. On the contrary, the urinary excretion of N-acetyl-β-glucosaminidase was higher (*P* = 0.001) in patients as compared with controls [46]. In a second longitudinal study, the authors observed a similar urinary excretion of N-acetyl-β-glucosaminidase before and after valproic acid for 6 months in 17 epileptic children [46]. Similarly, Korinthenberg et al. [41] found that valproic acid treatment for 3–4 months was not associated with an increased excretion of the mentioned enzyme in 14 children [41]. In contrast, the urinary excretion of N-acetyl-β-glucosaminidase increased (*P* < 0.01) after valproic acid for 8 months [40].

Unay et al. [45] assessed the excretion of N-acetyl-β-glucosaminidase in 46 treated children and found a higher (*P* < 0.01) urinary level of this enzyme in patients treated for more than 10 years. There was also a correlation (*P* < 0.01) between circulating valproic acid and the excretion of N-acetyl-β-glucosaminidase.

Verrotti et al. [42] measured the excretion of N-acetyl-β-glucosaminidase and β_2_-microglobulin in 23 children before as well as 6, 12, and 24 months after treatment. In these patients, valproic acid was associated with an increase (*P* < 0.01) in the excretion of N-acetyl-β-glucosaminidase. No association was noted with β_2_-microglobulinuria.

The excretion of β_2_-microglobulin and uric acid and the circulating level of uric acid were measured by Yoshikawa et al. [44] in ambulatory (*N* = 29) and non-ambulatory (*N* = 24) children on valproic acid. An increased excretion of β_2_-microglobulin (*P* < 0.02) and uric acid (*P* < 0.001) and low circulating level of uric acid (*P* < 0.0001) were observed in non-ambulatory as compared to ambulatory patients. Koga et al. [47] documented β_2_-microglobulinuria and circulating phosphate and uric acid in 87 patients on valproic acid. Univariate analyses revealed that a tubular injury was more likely to occur in non-ambulatory patients (*P* = 0.0001).

## Discussion

The present review of the literature can be recapitulated as follows: in patients with epilepsy, treatment with valproic acid for 7 months or more may sporadically induce an overt kidney tubular injury, which is sometimes associated with a decreased kidney function and usually resolves within 6 months after discontinuing this drug (without a difference between cases with and without carnitine); treatment for 7 months or more is often associated with a latent kidney tubular injury; the biochemical pattern of the overt kidney tubular injury and kidney biopsy studies point to the existence of a proximal tubular mitochondrial toxicity; the kidney tubular damage induced by valproic acid has so far been documented uniquely in patients with epilepsy (mostly associated with a severe developmental disability, non-ambulatory and concomitantly with two or more antiepileptic agents other than valproic acid); approximately every fifth case of overt valproic acid associated kidney tubular injury also presents with pathologically altered liver enzymes.

The discussion will focus on the mitochondrial toxicity of valproic acid, the mechanisms by which valproic acid induces a kidney tubular injury almost exclusively in severely disabled epileptics, and the therapy.

Valproic acid inhibits the mitochondrial ß-oxidation by reducing the intramitochondrial coenzyme A level, by inhibiting the involved enzymes, and by reducing circulating carnitine, an essential intermediary [3, 48, 49].

Three not mutually exclusive mechanisms might explain why valproic acid induces a kidney tubular damage almost exclusively in severely disabled epileptics. First, higher valproic acid doses might be necessary to treat seizures in these patients. Second, hypovitaminosis D, a cause of proximal tubular dysfunction [50], is common in disabled subjects with epilepsy [51]. Third, congenital mitochondrial diseases often affect the nervous system, sometimes the kidney, and occasionally both [52]. We speculate that some patients included in this analysis suffer from an undiagnosed mitochondrial disease with an overt nervous system involvement and a smoldering, biochemically asymptomatic tubular disease, which was unmasked by valproic acid.

The mainstay of therapy consists in discontinuing valproic acid. Supplementation with carnitine, vitamin D, or both may also be offered [3, 48].

The present review shows that valproic acid may cause both an overt and a pauci-symptomatic tubular injury. Hence, it may be speculated that the pauci-symptomatic injury represents the early stage, the overt injury the middle stage, and finally, the overt injury associated with a pathologically increased circulating creatinine the advanced stage of kidney damage induced by valproic acid. This hypothesis is supported by three cases included in the present survey [25, 37, 38].

The present analysis has some limitations. First, we found no more than about 50 individually documented cases of overt kidney tubular injury caused by valproic acid. Second, the reporting completeness was excellent only in every fifth case. Third, it has been suggested that therapy with carnitine may both avert and manage the kidney damage induced by valproic acid [3, 48]. However, we currently have no data to support or refute this recommendation. Fourth, available data do not allow to estimate the prevalence of the tubular damage caused by valproic acid, which is likely very uncommon. Future work is necessary to address the latter issues. In addition to the comprehensive literature search, the strength of this analysis is that it is the first known to systemically investigate the tubular injury induced by valproic acid.

Taken together, the results of the present systematic review point out that the biochemical abnormalities induced by valproic acid in patients with epilepsy strongly resemble those induced by some alkylating and platinating agents, by aminoglycoside antimicrobials, by the nucleotide reverse transcriptase inhibitors tenofovir and, more rarely, adefovir, and by the chelating agent deferasirox [6–8, 53]. The term de Toni-Debré-Fanconi syndrome has also been used to denote this kidney tubular injury.

In conclusion, valproic acid administered for 7 months or more in patients with epilepsy can rarely induce a proximal tubular injury, presenting with the typical features of de Toni-Debré-Fanconi syndrome. More frequently, a mild proximal tubular involvement, with isolated tubular proteinuria, mostly N-acetyl-β-glucosaminidase, has also been described.

## Supplementary information

Below is the link to the electronic supplementary material.

Graphical Abstract (PPTX 243 KB)

Supplementary file2 (DOCX 31 KB)

## Data Availability

The data supporting this study are available from the corresponding author upon reasonable request.
