# Supplementary material for: Kidney tubular injury induced by valproic acid: systematic literature review
Source: Pediatr Nephrol. 2023 Jan 16;38(6):1725–31. doi: 10.1007/s00467-022-05869-8 (PMC10154265; doi:10.1007/s00467-022-05869-8)
Supplement: Supplementary file 1 — Graphical Abstract (PPTX 243 KB) [file 467_2022_5869_MOESM1_ESM.pptx]

## Slide 1
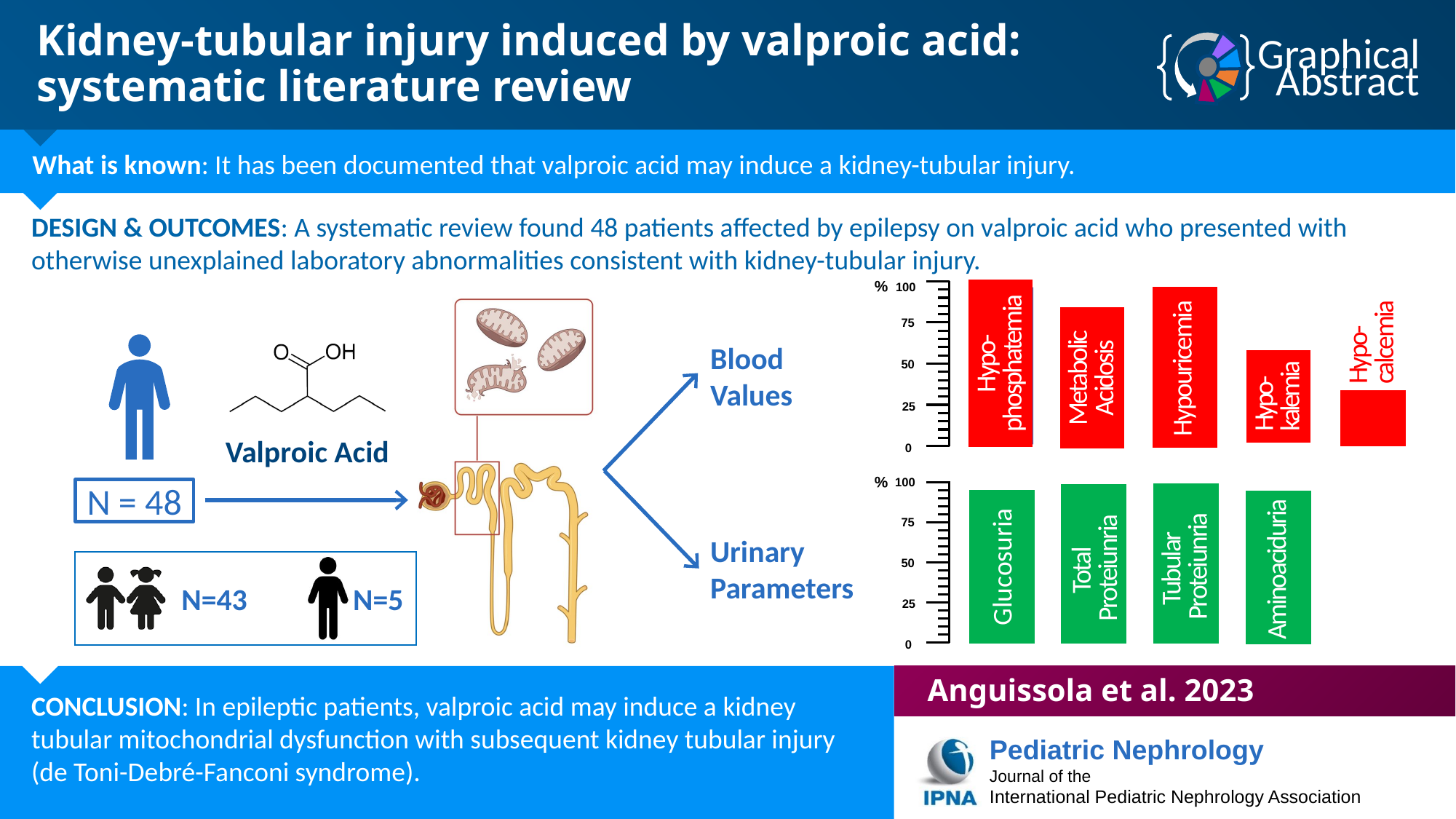

Kidney-tubular injury induced by valproic acid:
systematic literature review
What is known: It has been documented that valproic acid may induce a kidney-tubular injury.
DESIGN & OUTCOMES: A systematic review found 48 patients affected by epilepsy on valproic acid who presented with otherwise unexplained laboratory abnormalities consistent with kidney-tubular injury.
%
100
75
Hypo-
calcemia
Hypo-
phosphatemia
Blood
Values
Metabolic
Acidosis
Hypouricemia
50
Hypo-
kalemia
25
Valproic Acid
0
100
%
N = 48
75
Urinary
Parameters
Tubular
Proteiunria
Total
Proteiunria
Glucosuria
50
Aminoaciduria
N=43
N=5
25
0
Anguissola et al. 2023
CONCLUSION: In epileptic patients, valproic acid may induce a kidney tubular mitochondrial dysfunction with subsequent kidney tubular injury (de Toni-Debré-Fanconi syndrome).
